# Microencapsulated *Aliivibrio fischeri* in Alginate Microspheres for Monitoring Heavy Metal Toxicity in Environmental Waters

**DOI:** 10.3390/s141223248

**Published:** 2014-12-05

**Authors:** Dedi Futra, Lee Yook Heng, Salmijah Surif, Asmat Ahmad, Tan Ling Ling

**Affiliations:** 1 School of Chemical Sciences and Food Technology, Faculty of Science and Technology, Universiti Kebangsaan Malaysia, 43600 UKM Bangi, Selangor D.E., Malaysia; E-Mails: futra.dedi@yahoo.com (D.F.); salmij@ukm.my (S.S.); asmat@ukm.my (A.A.); 2 Southeast Asia Disaster Prevention Research Initiative (SEADPRI-UKM), LESTARI, Universiti Kebangsaan Malaysia, 43600 UKM Bangi, Selangor D.E., Malaysia; E-Mail: babybabeoo@gmail.com

**Keywords:** whole cell biosensor, heavy metal, optical fiber biosensor, *A. fischeri*, alginate microsphere

## Abstract

In this article a luminescence fiber optic biosensor for the microdetection of heavy metal toxicity in waters based on the marine bacterium *Aliivibrio fischeri* (*A. fischeri*) encapsulated in alginate microspheres is described. Cu(II), Cd(II), Pb(II), Zn(II), Cr(VI), Co(II), Ni(II), Ag(I) and Fe(II) were selected as sample toxic heavy metal ions for evaluation of the performance of this toxicity microbiosensor. The loss of bioluminescence response from immobilized *A. fischeri* bacterial cells corresponds to changes in the toxicity levels. The inhibition of the luminescent biosensor response collected at excitation and emission wavelengths of 287 ± 2 nm and 487 ± 2 nm, respectively, was found to be reproducible and repeatable within the relative standard deviation (RSD) range of 2.4–5.7% (*n* = 8). The toxicity biosensor based on alginate micropsheres exhibited a lower limit of detection (LOD) for Cu(II) (6.40 μg/L), Cd(II) (1.56 μg/L), Pb(II) (47 μg/L), Ag(I) (18 μg/L) than Zn(II) (320 μg/L), Cr(VI) (1,000 μg/L), Co(II) (1700 μg/L), Ni(II) (2800 μg/L), and Fe(III) (3100 μg/L). Such LOD values are lower when compared with other previous reported whole cell toxicity biosensors using agar gel, agarose gel and cellulose membrane biomatrices used for the immobilization of bacterial cells. The *A. fischeri* bacteria microencapsulated in alginate biopolymer could maintain their metabolic activity for a prolonged period of up to six weeks without any noticeable changes in the bioluminescence response. The bioluminescent biosensor could also be used for the determination of antagonistic toxicity levels for toxicant mixtures. A comparison of the results obtained by atomic absorption spectroscopy (AAS) and using the proposed luminescent *A. fischeri*-based biosensor suggests that the optical toxicity biosensor can be used for quantitative microdetermination of heavy metal toxicity in environmental water samples.

## 1. Introduction

Industrial and domestic wastewaters are being continuously released into the natural aquatic system every day. These wastewaters usually contain various heavy metal complexes. Zerovalent heavy metals are chemically inert, whilst metals with other oxidation states have strong biological activity and can induce genotoxic damage in living organisms [1–4]. The genotoxic metals cause damage to the genetic material in the cells via interaction with DNA sequences and structures or via breaking of dsDNA strands, resulting in mutagenic events such as chromosomal aberrations, changes in the structure and function of the reproductive system, reduction in growth rates and abnormal development in the embryo and adult stages of living organisms [3,5]. Furthermore, the negative impacts of heavy metals on human health have been suspected of causing cardiovascular diseases, reduction in intelligence, liver damage, *etc.* Therefore, there is a clear demand for sensitive and reliable assessment tools for heavy metal toxicity determination.

Toxicity bioassays using a commercial Microtox^®^ reagent containing freeze-dried culture of luminescent *A. fischeri* bacteria for heavy metal toxicity assay of contaminated water, soil and sediment, and measured with a laboratory-based or a portable photometer have been reported [6–9]. The Microtox^®^ bioassay is generally effective for detection of heavy metals in water samples, whereby it is able to distinguish between chemicals that are potentially hazardous and non-hazardous to microorganisms. However, the Microtox^®^ method possesses several drawbacks, e.g., low sensitivity, long response times (15 min to hours), high EC_50_ (50% effective concentration) values of >1 ppm and non-reproducible responses. Likewise, Tsiridis *et al.* [9] have carried out toxicity assays for Cu(II), Zn(II) and Pb(II) using a *A. fischeri*-Microtox^®^ reagent that exhibited long response times of up to 4 h. In addition, the luminescence response was observed to be unstable due to the free bacterial cells that were used, as the physically unsecured *A. fischeri* cells were susceptible to interference and gave erratic luminescence responses.

Other microbial heavy metal toxicity assays based on fluorescent or luminescent free bacterial cells using green fluorescent protein (GFP)-engineered *E. coli* [10,11], *E. coli* Alux gene [12] and *P. fluorescence* have been reported [13]. However, toxicity assays based on free bacterial cells in culture media could not determine heavy metal toxicity at low levels (<160 ppb) [13,14], and the incubation times are normally >2 h [10,12] before any changes to the luminescence signals are obtained [10].

To improve the bioassay performance for monitoring heavy metal toxicity, a biosensor would be an alternative method, whereby microbial cells are entrapped on a transducer to obviate any interference with enzyme activity, and offer assays that are highly sensitive, easy-to-use, with rapid exposure time and adaptable to online monitoring [15,16]. Microbial biosensors for heavy metal toxicity have been developed by using *A. eutrophos* Alux gene immobilized in alginate beads [17], *A. torulosa* alge entrapped in poly(2-hydroxyethyl methacrylate) hydrogels [18] and *E. coli* Alux gene immobilized in alginate beads [19]. However, these developed microbial biosensors had low sensitivity, high detection limits at the ppm level and long response times (15 min to 1.5 h).

In this work, we report the first compact sized biosensor for heavy metal toxicity investigation based on *A. fischeri* immobilized in alginate microspheres via microencapsulation and supported with a cellulose nitrate membrane. Alginate microspheres are commonly used as an enzyme immobilization matrix for designing biosensors such as glucose oxidase-based glucose biosensors [20], cholesterol oxidase for monitoring of cholesterol [21] and urease for examination of urea [22]. The proposed alginate microsphere-based biosensor detects heavy metal toxicity levels based on the electronic excitation of the bacterial bioluminescent reaction of the immobilized *A. fischeri* bacterial cells. *A. fischeri* is a naturally luminous bacterium that contains flavin reductase and luciferase biological catalysts. When the flavin reductase enzyme catalyzes the oxidation of reduced nicotinamide adenine dinucleotide phosphate [NAD(P)H] to NAD(P), flavin mononucleotide (FMN) is reduced (Equation (1)). The luciferase enzyme then further catalyzes the oxidation of reduced FMN (FMNH_2_), molecular oxygen and long-chain aldehyde to produce the corresponding FMN, water, long-chain carboxylic acid and a light quantum (Equation (2)), which is emitted at a wavelength of 490 nm [23,24].

(1) $$\text{NAD}(\text{P})\text{H}+{\text{H}}^{+}+\text{FMN}\overset{\text{flavin reductase}}{\to }\text{NAD}(\text{P})+{\text{FMNH}}_{2}$$

(2) $${\text{FMNH}}_{2}+{\text{O}}_{2}+\text{R}‐\text{CHO}\overset{\text{luciferase}}{\to }\text{FMN}+{\text{H}}_{2}\text{O}+\text{R}‐\text{COOH}+\text{Light}$$

As the microbial cells are exposed to toxic conditions caused by heavy metals, the cell metabolism is inhibited and and they give out a lower luminescence intensity, which can be registered instantaneously by a fiber optic spectrofluorimeter. Because a microsized alginate biomatrix was used to immobilize the biological component, the biosensor demonstrates rapid incubation times, high sensitivity, low LOD, good reproducibility and high stability.

## 2. Materials and Methods

### 2.1. Materials

All chemicals used were of analytical grade quality and dionized water was utilized for solution preparation. Stock solutions of 500 mg/L cadmium(II) chloride, zinc(II) chloride, copper(II) chloride, potassium dichromate (Sigma, St. Louis, MI, USA) were prepared by dissolving appropriate amounts of the respective salts in deionized water. Co^2+^, Ag^+^, Ni^2+^ and Fe^3+^ ion stock solutions at 700 mg/L were prepared by dissolving their nitrate salts (BDH, Radnor, PA, USA) in deionized water. Acid alginate (Sigma) stock solution in deionized water (6% w/v) was stored at 4 °C and can be used for over a month. Stock solutions of both 28 g/L nutrient agar (Scharlau, Bercelona, Spain) and 13 g/L nutrient broth (Oxoid, Hampshire, UK) containing 3% sodium chloride (Sigma) were prepared in deionized water and autoclaved at 121 °C for 20 min. All glassware used was cleaned by immersion in 15% nitric acid for 24 h to remove trace elements, followed by sterilization by autoclaving at 121 °C for 20 min. *A. fischeri* bacteria stock culture in 15% glycerol was obtained from the Microbiology Laboratory, Faculty of Science and Technology (National University of Malaysia, Malaysia) and kept at −80 °C.

### 2.2. Culture of A. Fischeri Bacteria

About 20 mL of *A. fischeri* bacteria stock culture in 15% glycerol was grown in 20 mL of nutrient agar medium at room temperature for 16 h. Single colonies of this bacterium were then isolated from the nutrient agar and grown in 4 mL of nutrient broth medium containing NaCl under shaking in a rotary thermo shaker at 250 rpm for 16 h at room temperature. Some 0.5 mL of this pre-cultured medium was then further grown in 50 mL of fresh NaCl-containing nutrient broth for 6 h under the similar condition. Then, the bacterial cells were harvested by centrifugation at 1000 rpm for 10 min followed by washing the *A. fischeri* cells with 3% NaCl for three times, and resuspended in 10 mL of 3% NaCl.

### 2.3. Fabrication of Microbial Toxicity Biosensors

To fabricate the alginate microsphere-based microbial biosensors, alginate microspheres were prepared via an emulsification technique. A mixture of alginate solution (1.5 mL, 2% w/v), liquid paraffin (4.5 mL, Sigma) and 2–3 drops of Tween 80 was mixed by strring on a magnetic stirrer at 900 rpm for 20 min to obtain an emulsion. About 1.5 mL of *A. fischeri* bacteria suspension in 3% NaCl was then added to the emulsion and uniformly mixed on a magnetic stirrer at 250 rpm for 10 min. Subsequently, by using a micropipette, the bacterial emulsion mixture was added dropwise into 0.15 M CaCl_2_-paraffin (2:1 v/v) under gentle stirring at <100 rpm. The *A. fischeri* encapsulated in alginate microspheres were collected by centrifugation at 1000 rpm for 10 min and washed thrice with sterile deionized water. The *A. fischeri* microencapsulated in alginate microspeheres was later filtered on a cellulose nitrate membrane (Whatman, 0.45 μm pore size and 5 cm diameter) using a Millipore vacuum pump, and left overnight at 4 °C in a refrigerator. The cellulose nitrate membrane with immobilized *A. fischeri*-encapsulated alginate microspheres was finally punched into a circular biosensing area of 19.63 mm^2^ by using an ordinary stationery paper punch. The bioluminescence response of the bacterial cell biosensor was measured with a Perkin Elmer fiber optic spectrofluorometer (Waltham, MA, USA) at excitation and emission wavelengths of 287 ± 2 nm and 487 ± 2 nm, respectively.

### 2.4. Effect of Cell Loading on the Bioluminescence Response

Different *A. fischeri* bacteria concentrations were prepared by culturing the cells in 50 mL of nutrient broth containing NaCl from 1–12 h. The concentrations of bacterial cells were measured using a Spectronic^R^ 20 Genesys™ UV-Vis spectrophotometer (Waltham, MA, USA) at the wavelength of 600 nm. The *A. fischeri* concentrations with optical densities (OD_600_) from 0.15 to 1.22 were then immobilized separately in alginate microspheres, and their bioluminescence responses were recorded at excitation and emission wavelengths of 287 ± 2 nm and 487 ± 2 nm, respectively.

### 2.5. pH Effect on the Whole Cell Biosensor Response

Toxicant solutions, e.g., Cu(II), Zn(II) and Pb(II) at various concentrations ranging from 0.01–500 mg/L were prepared in different pHs from pH 5.5–9.0 adjusted by utilizing 2 M NaOH and HCl. The *A. fischeri* bacterial cell concentration used was 28.8 mg/100 mL or 4.2 × 10^9^ CFU/mL (OD_600_ = 0.78−0.80). The luminescence response was recorded at room temperature 6 min after the initiation of the biochemical reaction.

### 2.6. Repeatability, Reproducibility and Stability Studies

Toxicity biosensors of *A. fischeri-*immobilized alginate microspheres made from alginate solution (2 % w/v), CaCl_2_ (0.15 M) and *A. fischeri* (4.2 × 10^9^ CFU/mL) were prepared, and their repeatability and reproducibility were determined using 20–1000 μg/L Cu(II), Cd(II), Pb(II) and Zn(II) in eight replicate measurements. For long term stability study, about 30 identical bacterial cell biosensors were prepared and kept at 4 °C. The stability of the biosensors was determined by measuring the luminescence intensity of the biosensors once a week for a period of ten weeks.

### 2.7. Effect of Individual Toxicant and Their Mixtures on the Biosensor Response

Prior to heavy metal toxicity exposure, the microbial biosensors were prehydrated with 20 μL of deionized water and incubated for 2 min for activation of the bacterial cells. Then, the activated biosensors was exposed to 30 μL of single toxicants [Cu(II), Cd(II), Pb(II), Zn(II) Cr(VI), Co(II), Ni(II), Ag(I) and Fe(III)] or toxicant mixtures [Cu(II), Cd(II), Pb(II) and Zn(II)] and allowed to react for 6 min. For the control experiment, 30 μL of deionized water was added instead. The biosensor response was investigated before and after exposure of the biosensor to the heavy metal toxicity. Each experiment was done in triplicate under the same experimental conditions. The relative luminescence unit percentage (% RLU) was calculated based on Equation (3) for single toxicants, whilst for toxicity mixtures, toxicity units (TU) were determined by using Equation (4), where A and B are two different heavy metals that coexist in the toxicant mixture, and EC_50_ is the effective concentration of a heavy metal at 50% of its relative luminescence intensity. When ΣTU = 1, it implies zero interaction additive effect between toxicants in a mixture. For ΣTU > 1, its additive index (AI) is estimated by AI = [ΣTU(−1) + 1], and this toxicity mixture is at an antagonistic level. ΣTU < 1, denotes a synergistic effect in the toxicant mixture, and its AI value can be calculated with AI = [(1/ΣTU) – 1] [25].

(3) $$\%\text{RLU}=\frac{\text{Luminescence intensity with analyte}}{\text{Luminescence intensity without analyte}}\times 100\%$$

(4) $$\Sigma^{TU}=\frac{A.EC_{50\%}\text{Mixture}}{A.EC_{50\%}\text{single}}+\frac{B.EC_{50\%}\text{Mixture}}{B.EC_{50\%}\text{single}}$$

### 2.8. Validation of the Biosensors for Monitoring River Water Toxicity

Four water samples were collected from the Langat River in Kajang, Malaysia and leachate was collected from the Jeram landfill in Mukim Jeram, Kuala Selangor, Malaysia using polyvinyl bottles. Then, the water samples were filtered using a cellulose nitrate membrane (0.45 μm pore size) and stored at 4 °C in a refrigerator. The microbial biosensor was then applied to determine heavy metal toxicity in the river water samples spiked with 20–100 μg/L Cu(II), 200–1000 μg/L Cd(II), 60–500 μg/L Pb(II) and 50–200 μg/L Zn(II), respectively, and validated by atomic absorption spectrometry (AAS, Perkin Elmer).

## 3. Results and Discussion

### 3.1. Characteristics of the Biosensor Luminescence Response

**Figure 1.**
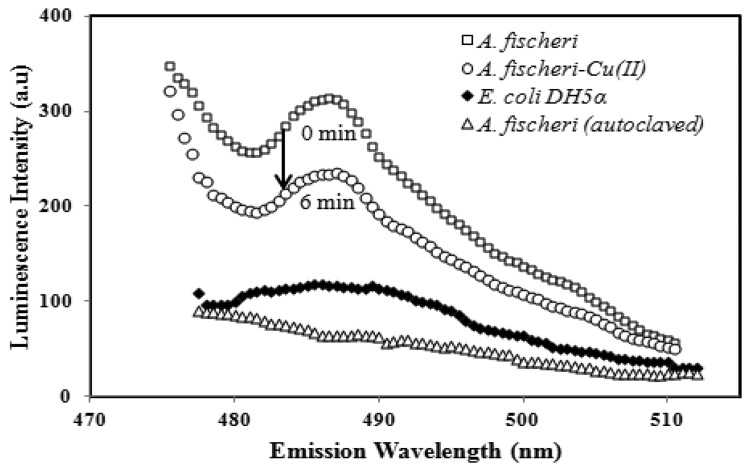
The luminescence response of the microbial biosensor before and after exposure to toxicant of 0.1 mg/L Cu(II), and comparison with luminescence response of immobilized DH5α *E. coli* and autoclaved *A. fischeri*.

The characteristics of the luminescent *A. fischeri* based-biosensor response before and after exposure to heavy metal toxicants and an emission spectra comparison with *E. coli* DH5α and autoclaved *A. fischeri* are illustrated in Figure 1.

In the absent of toxicants, the immobilized *A. fischeri* was found to give the highest lumimescence intensity at 487 ± 2 nm. The biosensor response at 487 ± 2 nm declined after incubation with 0.1 mg/L Cu(II) ion for 6 min, which was ascribed to the reaction of Cu(II) ion with the carboxylic acid functional groups of the bacterial cell, thereby inhibiting the cell metabolism. No luminescence responses were expected for either the non-fluorescent *E. coli* DH5α species and autoclaved *A. fischeri*, as the *A. fischeri* cells would have undergone sterilization, and were entirely destroyed.

### 3.2. Effect of the A. fischeri Cell Concentration

**Figure 2.**
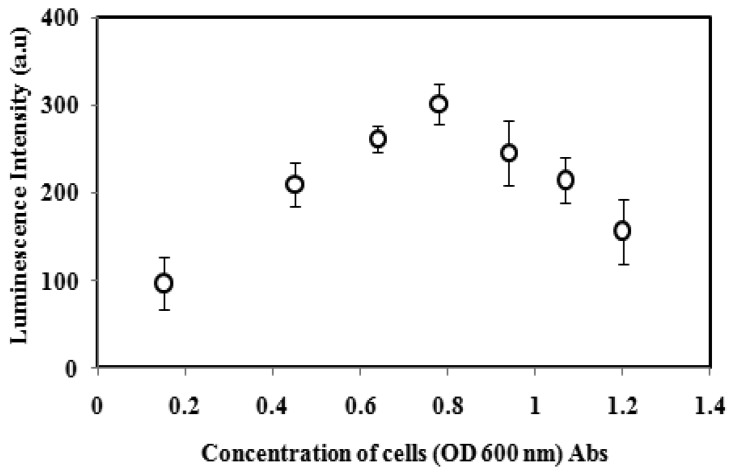
Luminescence intensity of the microbial biosensor at various *A. fischeri* cell concentrations from 0.15–1.22 OD_600_.

Optimization of bacterial cell loading in the alginate microspheres has been carried out in order to obtain the maximum luminescence intensity of the biosensor. As Figure 2 implies, the luminescence response of immobilized *A. fischeri* gradually increased with cell concentration from 0.15 to 0.78 OD at 600 nm due to the high rates of the enzymatic chemiluminescent rections. With further increases in the *A. fischeri* cell concentration from 0.94 to 1.22 OD, the luminescence response of the biosensor decreased because of the limited diffusion of oxygen into the high cell population alginate microspheres, which affected the metabolic activity of the bacterial cell for the normal production of DNA, RNA and enzymes [26,27]. Luminescence quenching of the microbial biosensor may also occur when too many bacterial cells are loaded into the alginate microspheres causing luminescence absorption by neighbouring *A. fischeri* cells and less energy to be emitted as a light quantum [27,28]. Therefore, the optimum cell concentration at OD of 0.78 to 0.80 (600 nm) was used for further experiments.

### 3.3. Effect of Toxicant Solution pH on the Biosensor Sensitivity

**Figure 3.**
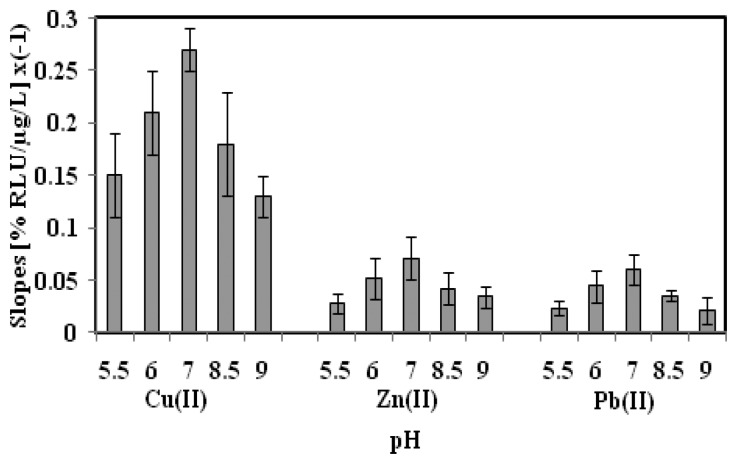
The sensitivity profile of biosensor based on *A. fischeri* encapsulated in alginate microspheres for toxicity investigation of Cu(II), Zn(II) and Pb(II) at pH 5.5–9.0.

The pH effect on the microbial biosensor sensitivity was investigated using Cu(II), Zn(II) and Pb(II) toxicant solutions at different pHs from pH 5.5 to pH 9.0 (Figure 3). The highest biosensor sensitivity was achieved at pH 7.0, whilst relatively low biosensor sensitivity was observed in slightly acidic and alkaline toxicant solutions. At lower pH conditions, *i.e.*, <pH 7.0, the chromophores of *A. fischeri* cells were protonated, whereby the carboxylic acid functional groups reacted with the abundant cationic metal ions and this interfered with the enzymatic chemiluminescent rections, thereby influencing the biosensor sensitivity [29,30]. At basic pH values, deprotonation of the chromophore resulted in amino acid chain disorder in the cells, and the bacterial cells were deactivated [31,32]. Thus, the toxicant solution pH was maintained at pH 7.0 for subsequent toxicity biosensing of heavy metals.

### 3.4. Repeatability, Reproducibility and Stability Characterizations

The response of the optical biosensor fabricated from *A. fischeri* encapsulated in alginate microspheres for monitoring of heavy metal toxicities was found to be repeatable and reproducible based on the promising relative standard deviations (RSDs) of <6% obtained (Table 1). This is due to the fact that the preparation technique via emulsification is able to produce uniform-sized alginate microspheres. The low repeatability and reproduciblity RDSs also suggest that the biosensor fabrication procedure is highly reproducible, and the biosensor can be reused for repetitive heavy metal toxicity assays (*n* = 8).

**Figure 4.**
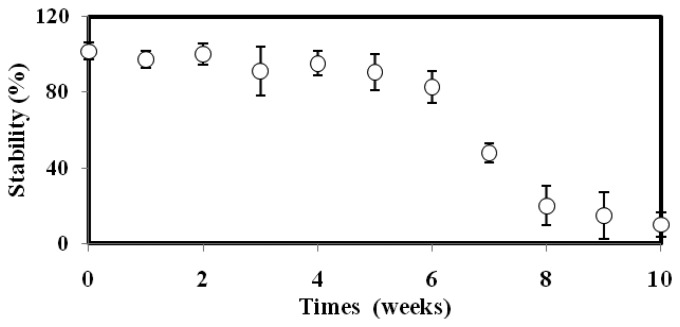
The stability of the biosensor for a testing period of 10 weeks.

The stability of the microbial biosensor as a function of time is portrayed in Figure 4. The biosensor maintained its 100% luminescence response for the first 5 weeks, and still managed to retain almost 90% of its original response in week 6. After that, the biosensor response was slowly reduced until the tenth week, when only a 10% bioluminescence response could be captured. This observation can be explained by the fact that the available nutrient sources for survival of the bacteria were getting reduced, and the enzyme was losing its chemiluminescent reaction activity. The toxicity biosensor based on *A. fischeri* encapsulated in alginate microspheres demonstrated higher stability compared to *Falvobacterium* sp. immobilized on glass fiber, where <80% of the biosensor stability was retained after 4 weeks of operation [33]. The high stability featured by the proposed *A. fischeri*-based biosensor was attributed to the alginate protection layer that rendered the bacterial cells less vulnerable to extreme temperatures and pH values.

### 3.5. Biosensor Response against Individual Heavy Metal Toxicity

**Figure 5.**
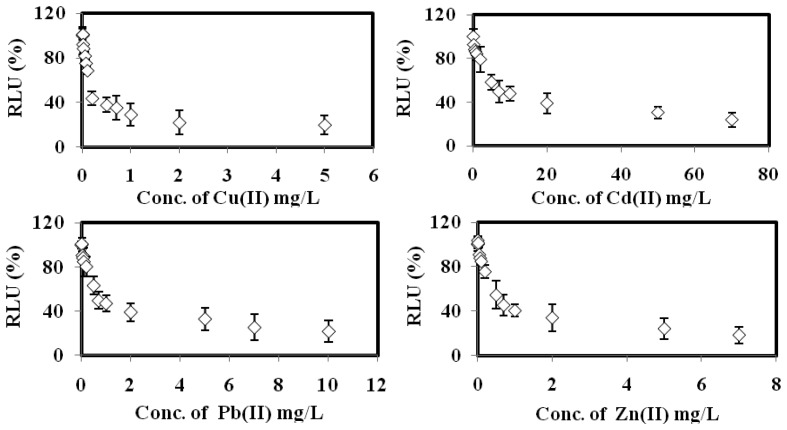

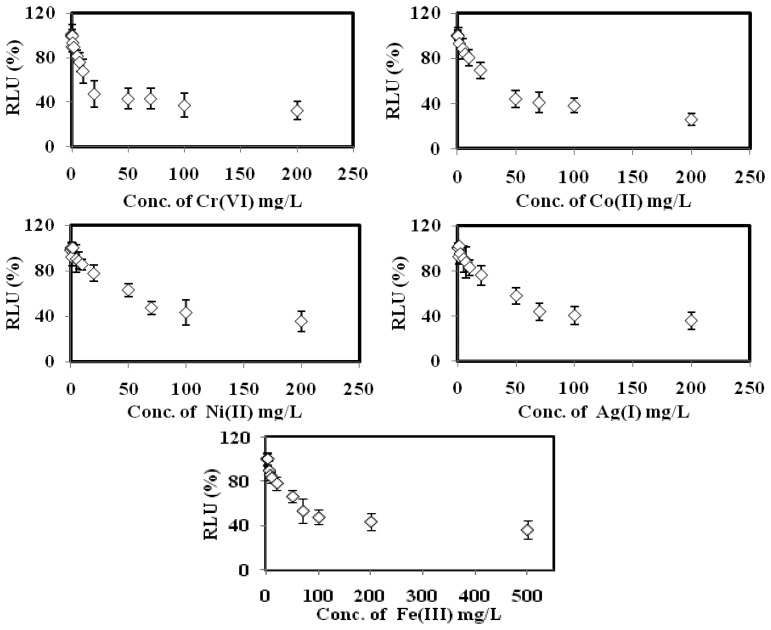
The bioluminescent sensor response towards Cu(II), Cd(II), Pb(II), Zn(II), Cr(VI), Co(II), Ni(II), Ag(I) and Fe(III) toxicities.

The bioluminescent sensor response towards the toxicities of Cu(II), Cd(II), Pb(II), Zn(II) Cr(VI), Co(II), Ni(II), Ag(I) and Fe(III), respectively, are displayed in Figure 5. The biosensor gave 100% RLU when it was free of toxicant. When the microbial toxicity biosensor exposed to the various concentrations of the respective heavy metals, the bacterial bioluminescent reaction was disrupted, and hence the bioluminescence signal was reduced with the increasing individual toxicant concentration [24,25].

Based on the data listed in Table 2, the toxicity optode based on *A. fischeri* demonstrated higher sensitivity towards Cu(II), Cd(II), Pb(II) and Zn(II) ions with 10–700 mg/L calibration range and limit of detection (LOD) below 0.32 mg/L. The relatively low EC_50_ values obtained for these heavy metal ions between 0.17 mg/L and 6.3 mg/L indicate that a small amout of these toxicants is sufficient to inhibit the microbial biosensor. The biosensor sensitivity sequence for heavy metal toxicity was found to be: Cu(II) > Zn(II) > Pb(II) > Cd(II) > Cr(IV) > Co(II) > Ag(I) > Ni(II) > Fe(III). Similar observations were obtained by Hoffman *et al.* [34] and Salizzato *et al.* [35], whereby the toxicity assay based on free *A. fischeri* cells demonstrated the highest sensitivity towards Cu(II) ion in environmental water samples after an incubation period of 30 min.

The toxicity biosensor made from *A. fischeri* encapsulated in alginate microspheres for fiber optical transduction of bioluminescent events shows better performance in terms of LOD, linear response range and response time when compared to amperometry [36], microplate reader luminometry [19] luminometry [37] and quartz cuvette spectroflourimetry [14,38] transducers using various types of unimmobilized recombinant microorganisms (Table 3).

Likewise for the toxicity assays using immobilized and unimmobilized *A. fischeri* cells coupled with microplate luminometry [37,40,41], amperometry [39,42], potentiometry [43] and spectrofluorimetry [44] transducers as well as a flow-through fluorescent sensor [45], as most of the previously reported toxicity assays involved long response times [37,40,41,44] and high microbial cell concentrations [45]. The EC_50_ values obtained for each heavy metal ion tested in this study using *A. fischeri*-immobilized alginate microspheres are compared with the reported EC_50_ values in Table 4. The EC_50_ values for Cu(II), Cd(II), Pb(II), Zn(II), Cr(VI) and Ni(II) ions from this study based on immobilized *A. fischeri* are found to be lower compared to the reported EC_50_ values for the respective metal ion toxicities based on unimmobilized *A. fischeri* bacteria [7], *P. viridis* mussel [46], *I. galbana* and *Synechococcus sp* algae [47] and *P. fluorescence* bacteria [48]. On the other hand, the reported EC_50_ values for Cu(II), Cd(II), Pb(II) and Zn(II) toxicities obtained by using *P. viridis* mussel [46], *I. galbana* and *Synechococcus sp.* algae [47], *P. fluorescence* bacteria [48], *D. magna* water flea [49], *A. fischeri* bacteria [50], *A. fischeri* and *J. lividum* (pUTluxAB) bacteria [6], *C. reinhardtii* and *P. subcapitata* algae [51], *P aeruginosa* bacteria [52], *A. tonsa* animalia [53], *Synechocystis* sp bacteria [54], *A. cepa* plant [55] and *L. quadridentata* algae [56] are appeared to be better then the EC_50_ values for the equivalent heavy metal toxicities using the proposed luminescent microbiosensor based on immobilized *A. fischeri*. However, the reported biotoxicity assays are very much dependent on free bacterial cells, long incubation times, high consumption of chemicals and the sensors are non-regenerable.

### 3.6. Effect of Toxicant Mixture on the Whole Cell Biosensor Response

For toxicity study using toxicant mixtures of Cu(II), Cd(II), Pb(II) and Zn(II) at various combinations and concentration ratios, the luminescent *A. fischeri* microoptode showed 100% antagonistic results (Table 5), which indicates antagonistic reactions occurred between toxic mixtures and the microbial cells. The different metal ions undergo intra-interactions before acting as Lewis acids to interact with the active sites of the bacterial cells (Lewis bases), and inhibit the cell metabolism reactions [7,57,58]. Consequently, lower toxicity levels are imparted to the bacteria cell.

As the bacterial cells experienced lower toxicity levels from the toxicant cocktails, a decrease in the the inhibition effect to immobilized *A. fischeri* cell resulted in a decrease of the EC_50_ values for the toxicant mixtures as compared to the EC_50_ values of the respective individual toxicants. Schmitz *et al.* [59] also reported about 90% antagonistic interactions in their toxicity assay using *Pseudmonas putida* dan *V fischeri* for Cd(II), Hg(II) and Pb(II) toxicity mixture detection. *Levidium sativum* and *Spirodela polyrrhiza* have also been used for antagonistic determination of Ni(II), Cr(VI) and Cu(II) toxic cocktails at 94% antagonistic level [60].

### 3.7. Biosensor Use for River Water Toxicity Evaluation

Tables 6 and 7 show the data for accordance between the results for Cu(II), Cd(II), Pb(II) and Zn(II) ions spiked into Langat River water and Jeram landfill leachate water samples by both the developed *A. fischeri*-based biosensor and the AAS method. The accordance values for the toxicity biosensor in the range of 82.7%–111.5% were found to be consistent with those of the AAS method (85%–108%). A statistical *t-test* based on the Miller and Miller method has been applied to compare the concentrations of Cu(II), Cd(II), Pb(II) and Zn(II) ions spiked in the Langat River water and Jeram landfill leachate obtained by using the optical toxicity biosensor and the conventional AAS method [61]. Based on the calculated *t* values (Tables 6 and 7), there were no significant differences between the metal ion concentrations determined by both methods. The results of the microbial biosensor based on *A. fischeri* was also in good agreement with AAS standard method for the determination of Cu(II), Cd(II), Pb(II) and Zn(II) ions in environmental water samples based on the satisfactory correlation coefficient (R^2^) values obtained in the range of 0.9956–0.9998 between the developed toxicity biosensor and AAS method (calibration slopes = 0.955–1.028).

However, further work is needed to improve the LOD values for Pb(II) and Cd(II) ions since the biosensor is intended to be applied for evaluation of environmental waters. This is because the limits for Pb(II) and Cd(II) ions' concentrations in drinking water advocated by the World Helth Organization (WHO) are 10 μg/L and 3 μg/L, respectively [62].

## 4. Conclusions

A toxicity biosensor based on *A. fischeri* immobilized in alginate microspheres has been successfully developed to detect heavy metal toxicity in environmental water samples. The bioluminescent toxicity biosensor is sensitive to heavy metal toxicity. It can be reused for toxicity evaluation at least 8 times and gives reproducible results. The microbial biosensor is highly stable and could be used to detect of heavy metals in environmental waters after short incubation times compared to common toxicity bioassay methods. The analytical performance of the biosensor is comparable with the conventional ASS method for heavy metal detection, therefore, the proposed toxicity biosensor based on *A. fischeri* has good potential to detect heavy metals in environmental water samples.

## Figures and Tables

**Table 1.** Repeatability and reproducibility RSDs for *A. fischeri*-based toxicity microbiosensor towards Cu(II), Cd(II), Pb(II) and Zn(II) toxicities.

**Heavy Metals**	**Concentrations (μg/L)**	**Repeatability *n* = 8, RSD (%)**	**Reproducibility *n* = 8, RSD (%)**
Cu(II)	20	2.4	5.6
	70	4.5	4.4

Cd(II)	500	5.7	4.7
	1000	5.4	5.3

Pb(II)	70	3.7	3.3
	200	3.2	2.8

Zn(II)	70	5.3	3.6
	200	5.5	4.9

**Table 2.** The bioluminescent microoptode performance towards Cu(II), Cd(II), Pb(II), Zn(II), Cr(VI), Co(II), Ni(II), Ag(I), and Fe(III) toxicities.

**Heavy Metal Ion**	**Dynamic Range (μg/L)**	**LOD (μg/L)**	**Slope(% RLU/μg/L)**	**EC_50_ (μg/L)**	**R^2^**
Cu(II)	(0.1 − 2) × 10^2^	6.40	−0.2512	1.7 × 10^2^	0.999
Cd(II)	(0.2 − 5) × 10^3^	1.56 × 10^2^	−0.0072	6.3 × 10^3^	0.998
Pb(II)	(0.5 − 7) × 10^2^	0.47 × 10^2^	−0.0601	7.0 × 10^2^	0.996
Zn(II)	(0.5 − 7) × 10^2^	0.32 × 10^3^	−0.0700	6.0 × 10^2^	0.996
Cr(VI)	(0.1 − 2) × 10^4^	0.10 × 10^4^	−0.0024	1.8 × 10^4^	0.992
Co(II)	(0.2 − 5) × 10^4^	0.17 × 10^4^	−0.0010	3.2 × 10^4^	0.939
Ni(II)	(0.5 − 7) × 10^4^	0.28 × 10^4^	−0.0006	6.6 × 10^4^	0.988
Ag(I)	(0.2 − 7) × 10^4^	0.18 × 10^4^	−0.0007	6.0 × 10^4^	0.986
Fe(III)	(0.5 − 7) × 10^4^	0.31 × 10^4^	−0.0005	7.0 × 10^4^	0.984

**Table 3.** Comparison of the developed toxicity biosensor performance with other reported toxicity biosensor for the determination of Cu(II), Cd(II), Pb(II), Zn(II), Cr(VI), Co(II), Ni(II), Ag(I) and Fe(III) toxicities.

**Heavy Metal**	**Biological Component**	**Immobilization Matrix**	**Dynamic Range (μg/L)**	**LOD (μg/L)**	**Time (min)**	**Reference**
Cu(II)	*A. fischeri*	Alginate microsphere	(10.0 − 200.0)	6.4	6	This work
	*S. cereviceae*	Agar gel Agarose	(3.4 − 26.9) × 10^4^	1.3 × 10^4^	8	[36]
	*E. coli (Alux)*	Carbon paste	(0.1 − 1.3) × 10^3^	0.1 × 10^3^	90	[19]
	C*ircinella* sp.	electrode	(6.7 − 134.0)	0.1	30	[39]

Cd(II)	*A. fischeri*	Alginate microsphere	(0.2 − 5.0) × 10^3^	156	6	This work
	*S. cereviceae*	Agar gel	(4.6 − 45.8) × 10^3^	1.8 x 10^3^	8	[36]
	*E. coli (Alux)*	Free culture	(0.1 − 1.0) × 10^2^	12	120	[37]

Pb(II)	*A. fischeri*	Alginate microsphere	(0.5 − 70.0) × 10^2^	47	6	This work
	*S. cereviceae*	Agarose	(6.9 − 417.0) × 10^3^	2.8 × 10^3^	8	[36]
	*E. coli* (Alux)	Free culture	(1.0 − 8.0) × 10^2^	12	30	[37]
	*E. coli* GFP	Free culture	(0.2 − 82.8) × 10^3^	0.2 × 10^3^	720	[14]

Zn(II)	*A. fischeri*	Alginate microsphere	(0.5 − 7.0) × 10^2^	32	8	This work
	*E. coli (Alux)*	Free culture	(0.03 − 8.0) × 10^5^	2.6 × 10^3^	120	[37]
	*E. coli (Alux)*	Free culture	(0.4 − 2.5) × 10^3^	0.4 × 10^3^	120	[38]

Cr(VI)	*A. fischeri*	Alginate microsphere	(0.1 − 2.0) × 10^4^	1.0 × 10^3^	6	This work
	*S. cerevisiae*	Agarose	(26 − 104) × 10^3^	10.4 × 10^3^	5	[36]
	*E. coli (luxAB)*	Free culture	-	2.0	15	[40]
	*A ferrooxidans*	Cellulose membrane	(0.02 − 118) × 10^3^	18.0	1	[41]

Co(II)	*A. fischeri*	Alginate microsphere	(2.0 − 50.0) × 10^3^	1.7 × 10^3^	6	This work
	*R. eutropha*	Free culture	(0.5 − 23.6) × 10^3^	0.5 × 10^3^	30	[42]

Ni(II)	*A. fischeri*	Alginate microsphere	(5.0 − 70.0) × 10^3^	2.8 × 10^3^	6	This work
	*R. eutropha*	Free culture	(0.2 − 17.5) × 10^2^	29.0	30	[42]
	*B. Sphaericus*	Whatman membrane	2.0 − 40.0	0.02	1.5	[43]

Ag(I)	*A. fischeri*	Alginate microsphere	(0.2 − 7.0) × 10^4^	1.8 × 10^3^	6	This work
	*E. coli* (lux)	Free culture	^-^	17	120	[44]

Fe(III)	*A. fischeri*	Alginate microsphere	(5.0 − 70.0) × 10^3^	3.1 × 10^3^	6	This work
	*P fluorescence*	Control pore glass	3.0 − 200.0	3.0	5	[45]
	*A ferrooxidans*	Cellulose membrane	(0.22 − 6050) × 10^2^	22.0	1	[41]

**Table 4.** Comparison of EC_50_ values for Cu(II), Cd(II), Pb(II), Zn(II), Cr(VI), Co(II), Ni(II), Ag(I) and Fe(III) toxicities obtained from the developed luminescent bacteria biosensor based on alginate microspheres with the EC_50_ values reported in the literatures.

**EC_50_ Value (μg/L)**									**Time (min)**	**Reference**

Cu(II)	**Cd(II)**	**Pb(II)**	**Zn(II)**	**Cr(VI)**	**Co(II)**	**Ni(II)**	**Ag(I)**	**Fe(III)**		
0.17 ×10^3^	0.63 × 10^4^	0.70 × 10^3^	0.60 × 10^3^	1.8 × 10^4^	6.6 × 10^4^	6.6 × 10^4^	6.0 × 10^4^	7.0 × 10^4^	6	This work
0.25 × 10^3^	0.74 × 10^3^	1.40 × 10^3^	-	-	-	-	-	-	1440	[47]
-	1.10 × 10^4^	-	0.86 × 10^3^	-	-	-	-	-	4320	[7]
4.20 × 10^3^	2.90 × 10^3^	4.20 × 10^3^	-	-	-	-	-	-	2880	[47]
4.40 × 10^4^	1.20 × 10^4^	-	0.65 × 10^2^	-	-	-	-	-	15	[48]
-	-	0.95 × 10^2^	0.30 × 10^3^	-	-	-	-	-	2880	[49]
-	-	-	-	1.2 × 10^4^	-	9.3 × 10^4^	7.9	-	15	[50]
-	-	-	-	7.5 × 10^3^	1.6 × 10^4^	2.7 × 10^2^	-	9.2 × 10^3^	15	[6]
-	-	-	-	-	-	-	2.0	-	360	[51]
-	-	-	-	-	1.5 × 10^2^	-	-	-	2880	[52]
-	-	-	-	-	-	0.3 × 10^2^	1.7 × 10^2^	-	2880	[53]
-	-	-	-	-	-	-	-	5.4 × 10^3^	5760	[54]
-	-	-	-	-	5.5 × 10^3^	-	-	-	5760	[55]
-	-	-	-	-	-	-	-	7.5 × 10^2^	2880	[56]

**Table 5.** The AI value and toxicity level of the toxicity biosensor for toxicant mixture determination.

**Toxicant Mixture**	**AI**	**Toxicity Rate**
(1:1 w/w)		

Pb(II) + Zn(II)	−2.716	Antagonistic
Cu(II) + Zn(II)	−0.892	Antagonistic
Cu(II) + Pb(II)	−0.867	Antagonistic
Cd(II) +Zn(II)	−0.397	Antagonistic
Cd(II) + Pb(II)	−0.906	Antagonistic
Cd(II) + Cu(II)	−0.271	Antagonistic

(2:1 w/w)		

Pb(II) + Zn(II)	−2.168	Antagonistic
Cu(II) + Zn(II)	−0.346	Antagonistic
Cu(II) + Pb(II)	−0.324	Antagonistic
Cd(II) +Zn(II)	−1.556	Antagonistic
Cd(II) + Pb(II)	−2.755	Antagonistic
Cd(II) + Cu(II)	−0.261	Antagonistic

(1:2 w/w)		

Pb(II) + Zn(II)	−1.499	Antagonistic
Cu(II) + Zn(II)	−1.032	Antagonistic
Cu(II) + Pb(II)	−0.795	Antagonistic
Cd(II) +Zn(II)	−0.782	Antagonistic
Cd(II) + Pb(II)	−0.517	Antagonistic
Cd(II) + Cu(II)	−2.450	Antagonistic

(1:1:1 to 1:1:1:1 w/w)		

Cu(II) + Cd(II) + Pb(II)	−2.811	Antagonistic
Cu(II) + Cd(II) + Zn(II)	−2.894	Antagonistic
Cd(II) + Pb(II) + Zn(II)	−1.285	Antagonistic
Cu(II) + Cd(II) + Pb(II) + Zn(II)	−5.447	Antagonistic

**Table 6.** Accordance of Cu(II), Cd(II), Pb(II) and Zn(II) ions spiked in Langat River water samples by both developed *A. fischeri*-based biosensor and AAS method.

**Heavy Metal**	**Added (μg/L)**	**Biosensor (*n* = 3)**		**AAS (*n* = 3)**		**Calculated t-test**

		**Found (μg/L)**	**Accordance (%)**	**Found (μg/L)**	**Accordance (%)**	
Cu(II)	0	17.71 ± 1.24	-	18.14 ± 1.32	-	0.317
	20	36.69 ± 3.20	94.90	37.58 ± 2.54	97.20	0.201
	50	59.04 ± 10.69	82.66	61.58 ± 4.14	86.88	0.509
	70	80.22 ± 10.47	89.30	83.28 ± 5.08	93.06	0.842
	90	103.85 ± 16.26	95.71	106.92 ± 8.06	98.64	0.416
	100	123.16 ± 22.51	105.45	120.68 ± 9.40	102.68	0.354

Cd(II)	0	303.86 ± 11.75	-	297.09 ± 1.58	-	0.959
	200	515.91 ± 20.20	106.03	503.83 ± 13.90	103.37	0.307
	500	785.30 ± 55.77	96.29	791.20 ± 36.77	98.82	0.238
	700	911.89 ± 90.78	86.86	946.72 ± 51.38	92.80	0.902
	900	1133.98 ± 112.11	92.23	1094.93 ± 68.28	88.65	1.249
	1000	1189.62 ± 116.51	88.58	1192.56 ± 81.68	89.54	0.366

Pb(II)	0	60.27 ± 1.51	-	61.63 ± 2.55	-	0.595
	60	112.38 ± 7.34	86.85	115.20 ± 4.66	89.28	0.770
	70	120.11 ± 18.57	85.49	126.37 ± 5.11	92.48	0.447
	100	158.67 ± 23.12	98.40	158.80 ± 9.30	97.17	0.114
	200	274.75 ± 41.61	107.24	271.48 ± 18.52	104.92	0.152
	500	578.98 ± 55.56	103.74	567.13 ± 35.48	101.10	1.035

Zn(II)	0	55.53 ± 1.75	-	54.71 ± 2.02	-	0.376
	50	103.93 ± 5.92	96.80	100.58 ± 4.31	91.74	1.100
	70	114.57 ± 12.39	84.34	116.08 ± 5.56	87.67	0.345
	90	152.73 ± 15.04	108.00	149.62 ± 8.68	105.46	0.249
	100	167.00 ± 21.09	111.47	161.85 ± 11.01	107.14	0.454
	200	265.94 ± 32.11	105.21	261.24 ± 15.83	103.26	0.142

Notes: The critical value, t_4_ = 2.78 (P = 0.05, 95%). The linear equation of [Cu(II)]_biosensor_ *versus* [Cu(II)_AAS_, [Cd(II)]_biosensor_ *versus* Cd(II)_AAS_, [Pb(II)]_biosensor_ *versus* [Pb(II)]_AAS_ and [Zn(II)]_biosensor_ *versus* [Zn(II)]_AAS_ were [Cu]_biosensor_ = 0.9723[Cu]_AAS_ – 2.805, [Cd]_biosensor_ = 1.0282[Cd]_AAS_ − 8.683, [Pb]_biosensor_ = 0.989[Pb]_AAS_ − 0.068 and [Zn]_biosensor_ = 0.9768[Zn]_AAS_ – 0.0541 respectively. the R^2^ values for Cu(II), Cd(II), Pb(II) and Zn(II) were 0.9968, 0.9956, 0.9993 and 0.9986, respectively. The linear equation used to determine of Cu(II), Cd(II), Pb(II) and Zn(II) concentration were (Y = −0.2604 + 95.063), (Y = −0.0066x + 93.751), (Y = −0.0610x + 92.174) and (Y = −0.0706x + 92.72), respectively.

**Table 7.** Accordance of Cu(II), Cd(II), Pb(II) and Zn(II) ions spiked in Jeram landfill leachate by both developed *A. fischeri*-based biosensor and AAS method.

**Heavy Metal**	**Added (μg/L)**	**Biosensor (*n* = 3)**		**AAS (*n* = 3)**		**Calculated t-test**

		**Found (μg/L)**	**Accordance (%)**	**Found (μg/L)**	**Accordance (%)**	
Cu(II)	0	11.59 ± 1.16	-	11.96 ± 0.96	-	0.307
	20	29.32 ± 2.73	88.65	30.43 ± 2.47	92.35	0.776
	30	36.76 ± 7.64	83.90	37.98 ± 2.61	86.73	0.280
	50	63.16 ± 6.19	103.08	61.83 ± 4.88	99.74	0.468
	70	85.50 ± 9.95	105.59	84.45 ± 6.11	103.56	0.328
	90	99.45 ± 13.03	97.62	97.03 ± 7.96	94.52	0.937
	100	108.39 ± 15.24	96.80	106.85 ± 8.04	94.89	0.452

Cd(II)	0	214.21 ± 3.30	-	215.103 ± 3.55	-	0.241
	200	411.35 ± 24.25	98.86	409.50 ± 14.45	97.20	0.388
	300	488.97 ± 33.28	91.57	484.47 ± 23.46	89.57	0.921
	500	641.49 ± 51.47	85.45	650.94 ± 42.52	87.17	0.238
	700	888.01 ± 68.54	96.26	879.60 ± 47.84	94.93	0.741
	900	1077.01 ± 95.22	95.86	1080.03 ± 47.89	96.10	0.023
	1000	1266.27± 129.12	105.21	1259.91 ± 83.81	104.58	0.069

Pb(II)	0	54.72 ± 1.44	-	55.19 ± 1.36	-	0.535
	60	104.89 ± 11.21	83.60	106.36 ± 4.41	85.28	0.217
	70	116.82 ± 13.37	88.71	118.22 ± 5.87	90.04	0.175
	80	138.22 ± 17.15	104.36	137.03 ± 6.51	102.30	0.137
	100	166.12 ± 21.95	111.39	163.48 ± 9.58	108.28	0.349
	200	251.55 ± 23.39	98.41	247.69 ± 16.52	96.25	0.262
	500	474.67 ± 67.12	94.93	532.43 ± 35.91	95.45	0.066

Zn(II)	0	64.28 ± 1.43	-	63.71 ± 0.87	-	0.479
	50	110.42 ± 6.71	92.28	110.72 ± 4.27	94.02	0.283
	70	131.78 ± 10.26	96.41	132.44 ± 5.51	98.18	0.262
	80	132.73 ± 13.65	85.56	133.45 ± 6.17	87.17	0.183
	90	142.02 ± 15.37	86.37	143.32 ± 7.15	88.45	0.192
	100	155.43 ± 18.25	91.15	157.62 ± 8.83	93.90	0.499
	200	274.13 ± 26.29	104.92	267.60 ±17.03	101.95	1.077

Notes: The critical value, t_4_ = 2.78 (P = 0.05, 95%). The linear equation of [Cu(II)]_biosensor_ *versus* [Cu(II)_AAS_, [Cd(II)]_biosensor_ versus Cd(II)_AAS_, [Pb(II)]_biosensor_ versus [Pb(II)]_AAS_ and [Zn(II)]_biosensor_ versus [Zn(II)]_AAS_ were [Cu] _biosensor_ = 0.9589[Cu]_AAS_ − 1.3885, [Cd]_biosensor_ = 0.9965[Cd]_AAS_ − 0.1318, [Pb]_biosensor_ = 1.0057[Pb]_AAS_ − 1.937 and [Zn]_biosensor_ = 0.9546[Zn]_AAS_ − 4.4235, respectively. The R^2^ values for Cu(II), Cd(II), Pb(II) and Zn(II) were 0.9996, 0.9996, 0.9998 and 0.9991, respectively.
